# Characterizing cortical responses to short-term multidisciplinary intensive rehabilitation treatment in patients with Parkinson’s disease: A transcranial magnetic stimulation and electroencephalography study

**DOI:** 10.3389/fnagi.2022.1045073

**Published:** 2022-11-03

**Authors:** Guangying Pei, Xinting Liu, Qiwei Huang, Zhongyan Shi, Li Wang, Dingjie Suo, Shintaro Funahashi, Jinglong Wu, Jian Zhang, Boyan Fang

**Affiliations:** ^1^School of Life Science, Beijing Institute of Technology, Beijing, China; ^2^Advanced Research Institute of Multidisciplinary Science, Beijing Institute of Technology, Beijing, China; ^3^School of Medical Technology, Beijing Institute of Technology, Beijing, China; ^4^School of Mechatronical Engineering, Beijing Institute of Technology, Beijing, China; ^5^Parkinson Medical Center, Beijing Rehabilitation Hospital, Capital Medical University, Beijing, China

**Keywords:** PD, TMS-EEG, primary motor cortex, beta oscillation, MIRT

## Abstract

Combined transcranial magnetic stimulation and electroencephalography (TMS-EEG) is a powerful non-invasive tool for qualifying the neurophysiological effects of interventions by recording TMS-induced cortical activation with high temporal resolution and generates reproducible and reliable waves of activity without participant cooperation. Cortical dysfunction contributes to the pathogenesis of the clinical symptoms of Parkinson’s disease (PD). Here, we examined changes in cortical activity in patients with PD following multidisciplinary intensive rehabilitation treatment (MIRT). Forty-eight patients with PD received 2 weeks of MIRT. The cortical response was examined following single-pulse TMS over the primary motor cortex by 64-channel EEG, and clinical symptoms were assessed before and after MIRT. TMS-evoked potentials were quantified by the global mean field power, as well as oscillatory power in theta, alpha, beta, and gamma bands, and their clinical correlations were calculated. After MIRT, motor and non-motor symptoms improved in 22 responders, and only non-motor function was enhanced in 26 non-responders. Primary motor cortex stimulation reduced global mean field power amplitudes in responders but not significantly in non-responders. Oscillations exhibited attenuated power in the theta, beta, and gamma bands in responders but only reduced gamma power in non-responders. Associations were observed between beta oscillations and motor function and between gamma oscillations and non-motor symptoms. Our results suggest that motor function enhancement by MIRT may be due to beta oscillatory power modulation and that alterations in cortical plasticity in the primary motor cortex contribute to PD recovery.

## Introduction

Parkinson’s disease (PD) is one of the most common neurodegenerative disorders and is characterized by different motor symptoms, such as akinesia, rigidity, and resting tremor (Weintraub et al., 2022). Neuroimaging studies have shown that cortical dysfunction contributes to the pathogenesis of motor symptoms in PD. The primary motor cortex (M1) is strongly implicated in the planning and execution of movements and converges the changes in the basal ganglia-thalamocortical circuit on motor impairment in PD (Leodori et al., 2022). Increasing M1 excitability by high-frequency repetitive transcranial magnetic stimulation (rTMS) has been demonstrated to improve motor symptoms (Yang et al., 2018). M1 transcranial direct current stimulation (tDCS) increases basal ganglia activity and normalizes cortical dysfunction, which plays a key role in motor skill learning (Horiba et al., 2019). Considering the superficial location of M1, which provides an effective and accessible target for therapeutic interventions, alterations in M1 excitability and plasticity may shed light on the mechanism of PD clinical treatments and their benefits on efficacy.

Oscillatory neuronal rhythms have been implicated in the pathophysiology of PD (Lee et al., 2021). TMS utilizes rapidly time-varying magnetic fields to sense electric fields in cortical tissue, resulting in non-invasive depolarization of cortical neurons (Rogasch et al., 2014). The combined use of transcranial magnetic stimulation and electroencephalography (TMS-EEG) allows direct recording of TMS-induced cortical activation with high temporal resolution and generates reproducible and reliable waves of activity without participant cooperation, representing a non-invasive tool qualifying the neurophysiological effects of interventions (Tremblay et al., 2019). TMS-EEG allows direct investigation of changes in local cortical excitatory and inhibitory circuits and reveals the connectivity between different cortical regions (Cao et al., 2021). The outcomes of TMS-EEG can be assessed by time domain with TMS-evoked potentials (TEPs), which are evoked following stimulation regions of the cortex. TEPs can be qualified by the global mean field power (GMFP), which measures the impact of the TMS pulse (Kerwin et al., 2018). In addition, analyzing the cortical oscillations in the time-frequency domain [i.e., the TMS-evoked oscillatory response (EOR)] has been associated with specific behaviors or cognitive functions in specific cortical or subcortical regions (Thut and Miniussi, 2009). Numerous studies have shown that TMS-EEG can be used to interrogate the physiology of the stimulated region and broader cortical activity, which complements structural and functional neuroimaging studies and provides further fundamental insights from a neurophysiological research perspective.

Although research remains limited, several recent studies have begun to emphasize the utility of TMS-EEG in characterizing patterns of brain activity in PD. Dynamic changes in the P60 component of TEPs suggest that M1 is likely a converging node in the network that generates re-emergent tremor (Leodori et al., 2020). Levodopa intake resulted in a significant increase in cortical excitability near supplementary motor areas (SMAs) in PD, as measured by TMS-EEG (Casarotto et al., 2019). The characterization of TEPs measured by TMS-EEG over M1 and the dorsal lateral prefrontal cortex (DLPFC) showed higher cortical excitability along with increased variability and lower widespread evoked potentials in PD (Maidan et al., 2021). As a powering technology, TMS-EEG contributes to the understanding of abnormal M1 in PD and has considerable potential for the study of the underlying mechanisms of clinical interventions.

Levodopa is the primary treatment for PD, and deep brain stimulation (DBS) is a common surgical treatment for PD symptoms that modifies abnormal electrical activity in the brain (Lee et al., 2021). Despite advances in pharmacology and surgery, no cure is available for PD. Considering the complex medical and rehabilitation needs of people with PD, multidisciplinary intensive rehabilitation treatment (MIRT) is a promising field for investigation. Several studies have shown that PD patients have a better response to MIRT (Frazzitta et al., 2013, 2014). MIRT sessions often consist of physical therapy, exercise, and even medication, and the diverse targets complicate determination of the optimal intervention time for PD. Despite MIRT traditionally taking 4 weeks or more, our previous study found that 2 weeks of short-term MIRT can still effectively improve main motor functions in PD patients (Chen et al., 2021). However, to the best of our knowledge, the neural mechanism of the beneficial effect of MIRT on PD is still unclear. Previous TMS-EMG studies have shown that both levodopa and DBS can act on the M1 intracortical inhibitory circuit of the motor cortex (Hanajima et al., 2004). Only one TMS-EEG study revealed modulation of early and later TEP components by subthalamic nucleus DBS (STN-DBS) and levodopa, respectively (Casula et al., 2017). Pathological neuronal synchronization of fast frequency bands in PD may be a causal factor in motor and cognitive impairment. However, no TMS-EEG research has been conducted to explore the effects of MIRT on TMS-related potentials and oscillations.

This study aimed to examine changes in cortical activity and neural oscillations after a short-term MIRT program by using single-pulse TMS over M1. We first recorded TMS-EEG in PD patients before and after MIRT. According to the treatment effects on primary motor symptoms, the TMS-evoked EEG dynamics were evaluated in MIRT responders and non-responders separately. Given that previous studies have shown that exercise improves neuroplasticity and modulates beta-band power to improve motor symptoms following PD neurofeedback (Khanna et al., 2017), we hypothesized that MIRT responders would exhibit significant changes in cortical activity and beta-band oscillations with TMS-EEG over the M1 by MIRT.

## Materials and methods

### Subjects

Forty-eight PD patients (61.94 ± 6.97 years) were admitted to the Neurological Rehabilitation Center of Beijing Rehabilitation Hospital from July to November 2020. Inclusion criteria included (1) Hoehn-Yahr (H-Y) Stage: II-III; and (2) an ability cooperate with treatment and evaluation. The exclusion criteria were (1) severe resting tremors; (2) Parkinson’s syndrome; (3) metal implants in the body, such as cardiac pacemakers and brain pacemakers; (4) a history of epilepsy; and (5) serious diseases of other systems.

### Trial design and treatment

**FIGURE 1:**
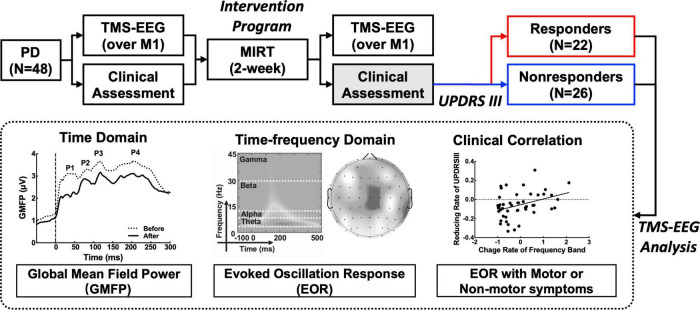
Workflow of the Parkinson’s disease (PD) treatment design and transcranial magnetic stimulation and electroencephalography (TMS-EEG) analysis.

All participants underwent the short two-week MIRT program mentioned in our previous study (Chen et al., 2021), including four daily rehabilitation sessions in the hospital. MIRT consisted of four daily sessions 5 days per week, and the duration of every single session varied from 30 to 60 min. The first session was one-on-one physical treatment. The second session was balance and gait exercises. The third session was aerobic training, and the fourth session was speech therapy. Participants were assessed for clinical symptoms, and TMS-EEG data were collected before and after MIRT. The study was approved by the Ethics Committee of Beijing Rehabilitation Hospital, Capital Medical University (2020bkky010), and all participants signed an informed consent form before inclusion. All patients were in a stable medication state with no medication adjustments during MIRT and follow-up. The workflow of the trial is shown in Figure 1.

### Clinical assessment

The primary outcome of motor symptoms was measured by the Movement Disorder Society Unified PD Rating Scale part III (MDS-UPDRS III), which includes four subscale scores for rigidity, tremor, axial, and bradykinesia. The secondary motor symptoms assessment included the Modified Parkinson Activity Scale (M-PAS), Five Times Sit to Stand (FTSTS), Berg Balance Scale (BBS), Timed Up and Go (TUG), 6-Minute Walk Distance (6MWD), and 10-Meter Walking (10MW). The non-motor outcomes were changes in the Mini Mental State Examination (MMSE), Montreal Cognitive Assessment (MoCA), Stroop Color-Word Test, and Paced Auditory Serial Addition Task (PASAT). The 39-Item PD Questionnaire (PDQ-39) was reassessed through a 3-month follow-up (Chen et al., 2021).

### Transcranial magnetic stimulation and electroencephalography recording and preprocessing

TMS was conducted by a rapid magnetic biphasic stimulator equipped with a figure-8 coil (70 mm) (Magstim Company Limited, Whitland, UK). The TMS stimulation locations were M1 of the left hemisphere. Eighty single-pulse stimuli of TMS were applied at the left primary motor cortex (M1) with an interval of 2 to 4 s (Koch et al., 2019). The intensity of TMS was 90% of the resting motor threshold (Rothwell, 1997). The EEG signals were recorded continuously using a 64-channel system (ANT Neuro GmbH, Germany). The impedance of the electrodes was kept below 5 kΩ. EEG data were preprocessed using a fully automated TESA toolbox (Rogasch et al., 2022) in MATLAB (MathWorks Inc., Natick, MA, USA). Preprocessing included bandpass filtering of 0.1-100 Hz and band-stop filtering of 48-52 Hz, TMS decay, and artificial noise removal. The first round of independent component analysis (ICA) was performed to remove voltage decay artifacts caused by scalp muscle activation and TMS-induced EMG. The second round of ICA was used to remove residual non-TMS lock-in artifacts, including blinking, horizontal eye movements, continuous muscle activity, and electrode noise. Finally, the preprocessed TMS-EEG signals were rereferenced to a common average reference. The original EEG signal can be purified into clean EEG signals by preprocessing.

### Time domain analysis

The GMFP was used to assess overall activity in the cerebral cortex evoked by TMS. GMFP represents the standard deviation (SD) of all the electrodes on the scalp, as shown in Formula (1) (Ozdemir et al., 2021), where *k* represents the total number of channels, *i* is the serial number of channel-I, *V_*i*_(t)* is the EEG amplitude of the *i-th* channel, and *V*_*mean(t)*_ is the average amplitude across all channels at time *t*. In this study, the GMFP of each subject was calculated at every time point from 300 ms before to 500 ms following a single TMS pulse. For each participant, the first four peaks (P1, P2, P3, and P4) of the GMFP waveform were detected within 300 ms following the TMS pulse over M1.


(1) $$G\,M\,F\,P=\sqrt{\frac{\sum_{i}^{k}{\left(V_{i}\,\left(t\right)\;-\;V_{m\,e\,a\,n}\,\left(t\right)\right)}^{2}}{k}}$$

### Time-frequency domain analysis

The EEG data were segmented with time windows from 300 ms before to 500 ms after the TMS pulse, and time/frequency decomposition was performed for each segment of EEG based on the Morlet wavelet (parameter = 7) (Zhang et al., 2022). The spectral power of interest was averaged in a time window from 100 ms to 400 ms and was extracted as follows: theta (4-7 Hz), alpha (8-13 Hz), beta (14-30 Hz), and gamma (31-45 Hz). TMS-evoked spectral power was averaged separately for each frequency band in each channel to assess global oscillatory activity.

### Statistical analysis

Statistical analysis was performed using the applied statistical software IBM SPSS Statistics 22.0 (SPSS Inc., Chicago, IL, United States) and MATLAB (MathWorks Inc., Natick, MA, USA). The demographic and clinical scale scores were tested for a normal distribution using a paired sample *T*-test or the independent sample *T*-test; otherwise, the Wilcoxon signed rank sum test or the Mann-Whitney *U* test was used. Paired sample *T*-tests were used for the GMFP within groups. The Wilcoxon signed rank test was performed on each kind of oscillatory power (theta, alpha, beta, and gamma) at each electrode in the whole brain within the group before and after MIRT. Correlations between clinical (UPDRS III, MMSE, and PDQ-39) and neurophysiological data (oscillatory power with a significant difference after MIRT) were tested by Spearman’s coefficients. All *p* -values were corrected with a false discovery rate (FDR).

## Results

### Effects of multidisciplinary intensive rehabilitation treatment on clinical characteristics

According to the results of the minimal clinically important difference (MCID) threshold of the MDS-UPDRS III (MDS-UPDRS III_*after*_-MDS-UPDRS III_*before*_ < −3.25) (Horvath et al., 2015), subjects were divided into the responding group and the non-responding group of MIRT. No significant difference in clinical characteristics at baseline (before MIRT) was found between the two groups (Supplementary Table 1). In the responding group, significant improvements in both motor and non-motor symptoms were observed after MIRT. Motor improvements were mainly reflected in the MDS-UPDRS III (*t* = 6.156, *p* < 0.001), bradykinesia subscale (*t* = 5.605, *p* < 0.001), axial subscale (*t* = 2.667, *p* = 0.014), M-PAS (*z* = −2.572, *p* = 0.010), BBS (*z* = −3.358, *p* = 0.001), and 6MWD (*z* = −2.728, *p* = 0.006). Non-motor symptoms were mainly reflected in the MMSE (*z* = −2.839, *p* = 0.005), MoCA (*z* = − 3.423, *p* = 0.001), Stroop test (*z* = −2.038, *p* = 0.042), PASAT (*t* = −3.179, *p* = 0.005) and 3-month effect in the PDQ-39 (*z* = −3.329, *p* = 0.001). However, in the non-responding group, the significant improvements were mainly in non-motor symptoms, especially those evaluated by the MMSE (*z* = −3.035, *p* = 0.002), MoCA (*z* = −3.693, *p* < 0.001), Stroop test (*z* = −3.156, *p* = 0.002) and PASAT (*t* = −4.229, *p* < 0.001). However, the TUG scores (*z* = −2.806, *p* = 0.005) showed significant enhancement after MIRT (Table 1).

**TABLE 1:** Clinical characteristics of the study groups.

PD		Responding group (*N* = 22)		Non-responding group (*N* = 26)			
		Before MIRT mean (SD)	After MIRT mean (SD)	*P*-value	Before MIRT mean (SD)	After MIRT mean (SD)	*P*-value
Motor symptoms assessment	MDS-UPDRS III	43.09(10.88)	35.27(12.74)	**<0.001** ^a^	37.12(10.62)	38.19(11.57)	0.142^a^
	Tremor	4.46(4.00)	3.82(3.50)	0.081^b^	3.77(3.67)	4.31(4.41)	0.097^b^
	Bradykinesia	21.82(7.10)	16.59(7.97)	**<0.001** ^a^	19.08(6.12)	19.42(6.26)	0.670^b^
	Rigidity	9.86(1.98)	9.14(2.12)	0.150^b^	8.89(1.73)	9.27(2.11)	0.187^a^
	Axial	6.96(3.74)	5.73(3.04)	**0.014^a^**	5.39(2.58)	5.19(2.93)	0.227^b^
	M-PAS	49.68(6.85)	51.14(8.68)	**0.010^b^**	51.54(5.27)	52.31(4.15)	0.179^b^
	FTSTS	10.89(2.37)	10.13(2.24)	0.103^a^	10.73(2.56)	11.01(2.85)	0.628^a^
	TUG	9.75(2.24)	9.35(2.07)	0.334^a^	10.90(5.39)	9.17(1.94)	**0.005^b^**
	BBS	22.68(3.56)	24.64(3.19)	**0.001^b^**	23.42(2.27)	24.46(2.87)	0.064^b^
	10MW-Com	1.25(0.22)	1.27(0.16)	0.623^a^	1.14(0.25)	1.17(0.20)	0.309^b^
	10MW-Fast	1.74(0.29)	1.74(0.31)	0.924^a^	1.56(0.35)	1.60(0.26)	0.269^b^
	6MWD	448.32(112.85)	477.96(112.04)	**0.006^b^**	436.46(87.54)	451.32(90.97)	0.054^b^
Non-motor symptoms assessment	MMSE	27.09(2.67)	28.68(2.15)	**0.005^b^**	27.31(2.88)	28.58(1.88)	**0.002^b^**
	MoCA	24.95(3.33)	27.00(3.12)	**0.001^b^**	25.08(4.26)	27.52(3.28)	**<0.001^b^**
	Stroop Test	45.09(17.07)	52.09(2.64)	**0.042^b^**	50.27(4.60)	52.31(3.08)	**0.002^b^**
	PASAT	23.50(16.58)	28.41(13.01)	**0.005^a^**	21.50(14.95)	29.00(15.82)	**<0.001^a^**
3-month	PDQ-39	24.14(10.12)	16.05(9.87)	**0.001^b^**	24.03(9.16)	20.61(10.53)	0.113^a^

Values are the mean (SD).

SD, Standard Deviation; MIRT, Multidisciplinary intensive rehabilitation treatment; MDS-UPDRS III, Movement Disorders Society-Sponsored Revised Unified Parkinson’s Disease Rating Scale part III; M-PAS, Modified Parkinson’s Activity Scale; BBS, Berg Balance Scale; FTSTS, Five Times Sit to Stand; TUG, Timed Up and Go; 10MW, 10-Meter Walking; 6MWD, 6-Min Walking Distance; PDQ-39, 39-Item Parkinson’s Disease Questionnaire; MMSE, Mini Mental State Examination; MoCA, Montreal Cognitive Assessment; PASAT, Paced Auditory Serial Addition Task.

^a^Paired samples *T*-tests.

^b^Wilcoxon signed-rank tests. *P*-values with significant differences are bolded.

### Transcranial magnetic stimulation-evoked cortical response

**FIGURE 2:**
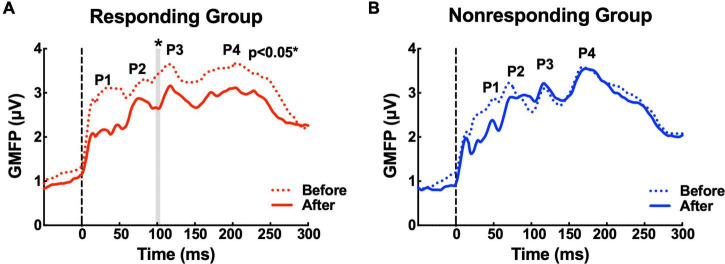
The waveforms of the global mean field power (GMFP) before and after multidisciplinary intensive rehabilitation treatment (MIRT). **(A)** The solid and dashed red curves depict GMFP in the responding group before and after MIRT. The gray shaded area shows a significant difference in GMFP amplitude before and after MIRT in the responding group. *p < 0.05 with correction. **(B)** The solid and dashed blue curves depict GMFP in the non-responding group before and after MIRT.

The GMFP amplitude evaluated over the whole brain was lower for both groups after MIRT compared to that at baseline (before MIRT). A significant reduction from 98 ms to 104 ms (104 ms: *p* = 0.046 with correction) was identified by a point-to-point paired *T*-test, which was mainly located between the P2 and P3 peaks, following the TMS pulse over M1 in the responding group (Figure 2A). However, no significant difference in the GMFP amplitude was noted in the non-responding group (Figure 2B).

### Transcranial magnetic stimulation-evoked oscillation response

**FIGURE 3:**
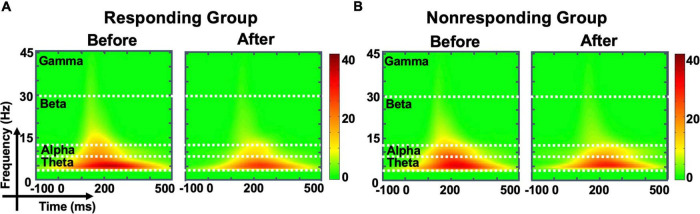
The evoked oscillatory power in the whole brain. The time-frequency plot shows the average energy for all electrodes in the whole brain before and after multidisciplinary intensive rehabilitation treatment (MIRT) in responding **(A)** and non-responding groups **(B)**.

**FIGURE 4:**
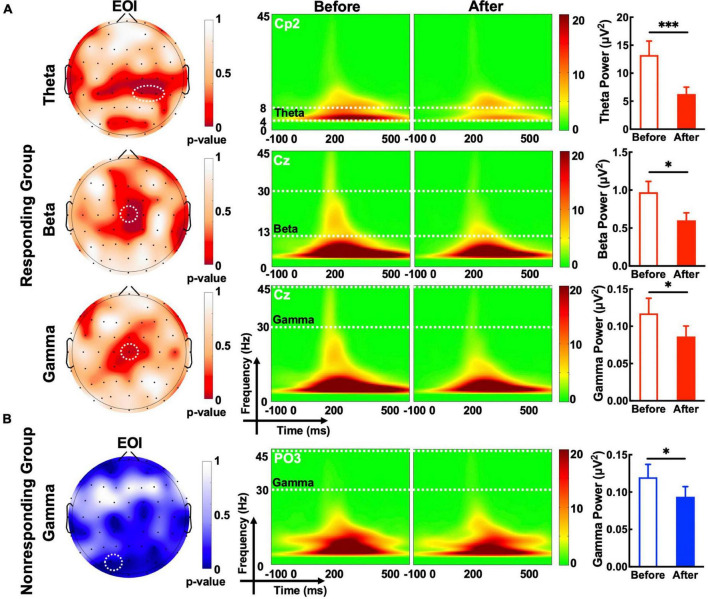
The evoked oscillatory power in the electrode of interest (EOI). **(A)** The topographic map shows a significant difference in the *p*-value in the target frequency band before and after MIRT. White dashed circles denote electrodes with significant differences (*p*-values corrected); Theta EOIs: Cp2 and Cp4; Beta EOI: Cz; Gamma (responding group) EOI: Cz; Gamma (non-responding group) EOI: PO3. **(B)** The time-frequency plot and histogram show the target frequency band power recorded from EOI before and after MIRT. *p < 0.05, ^***^p < 0.001 with correction.

Compared with observations before MIRT, the mean power of each frequency band (theta, beta, alpha, and gamma, all *p* -values >0.05) across all electrodes in the whole brain did not show significant changes in either responders or non-responders within the group or across the group after MIRT (Figure 3) (all *p* -values >0.05). However, after 2 weeks of MIRT, significantly decreased power was observed in the theta band (Cp2 electrode: *z* = −3.652, *p* < 0.001 and Cp4 electrode: *z* = −2.971, *p* = 0.024), beta band (Cz electrode: *z* = −2.776, *p* = 0.024), and gamma band (Cz electrode: *z* = −2.062, *p* = 0.039) in the responders mostly in the right parietal and central areas (Figure 4A), while only the gamma frequency band power was lower in the non-responders at the PO3 electrode (*z* = −2.933, *p* = 0.045) following TMS over M1 (Figure 4B). No significant changes in the oscillatory power in the alpha band of any electrodes were observed in either group (all *p* -values >0.05).

### Correlations between clinical and neurophysiological data

**FIGURE 5:**
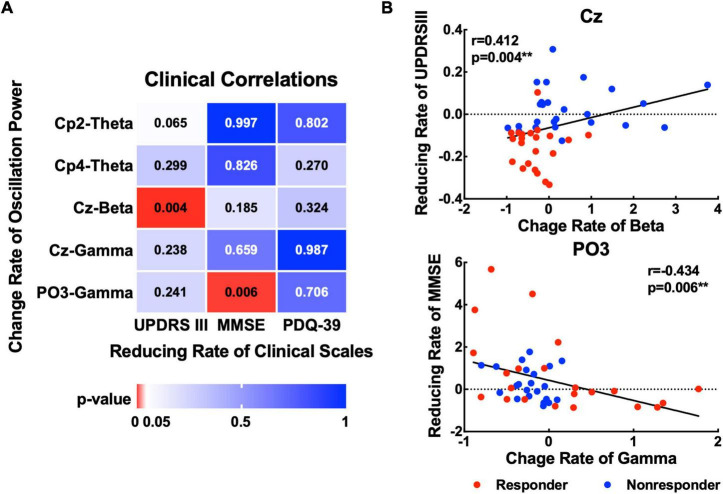
Association between changes in oscillatory power and clinical scales. **(A)** The *p*-values for the correlations between the change rate of the oscillatory power (theta, beta, and gamma) with significant changes and three kinds of clinical scales (UPDRS III, MMSE, and PDQ-39). **(B)** Scatter plots show significant correlations between the rate of change of beta power in the Cz electrode and the rate of change of gamma power in the PO3 electrode with MMSE. **p < 0.01 with correction.

To explore the association between the EOR and motor or non-motor symptom effects on MIRT. Correlation analysis of the change rate before and after MIRT was performed for MDS-UPDRS III, MMSE, and PDQ-39 scores with significant oscillatory power recorded from the EOIs (Figure 5). A positive correlation was found between the change rate of beta power recorded from the Cz electrode and UPDRS III scores (*r* = 0.412, *p* = 0.004). A negative correlation was identified between the rate of change in gamma power at the PO3 electrode and MMSE scores (*r* = −0.434, *p* = 0.006). The change rate of theta power recorded from Cp2 or Cp4 electrodes was not significantly correlated with any of the three clinical scales (all *p* -values >0.05).

## Discussion

In this study, we examined changes in time and time-frequency domain activity by TMS-evoked TEP with GMFP and oscillatory power of PD subjects before and after short-term MIRT. Our results reveal decreased GMFP amplitude and reduced oscillatory power within the theta, beta, and gamma bands in the MIRT responders but showed only decreased oscillatory power within the gamma band in the non-responders. Furthermore, the attenuated beta power following the TMS pulse over the primary motor cortex (M1) was significantly associated with the decreased UPDRS III score, and the increased MMSE score was strongly related to lower gamma power.

Previous studies have reported that the ability of M1 macrophages to functionally alter in response to stimuli is impaired in PD, which varies with disease severity. The GMFP results of this study showed that the responders showed a significant decrease at a global level in temporal windows approximately 100 ms after MIRT. These results are consistent with the neuroimaging findings of higher cortical excitability and variability in PD patients than in healthy people, which was explained by compensatory cortical reorganization (Wu et al., 2009). Hyperactivation in subcortical and cortical regions has been typically associated with inhibitory control in PD patients (Paz-Alonso et al., 2020). Upper limb rigidity is directly related to hyperactivation in the contralateral M1 (Yu et al., 2007). Intermittent theta burst (iTBS) stimulation to the M1 decreased GMFP by reducing cortical inhibition (Bai et al., 2021). A meta-analysis based on eight randomized controlled trials (RCTs) suggested that low-frequency rTMS could exert a significant effect on motor function in PD patients (Zhu et al., 2015). Overall, our findings support the proposal that cortical plasticity can be modulated by MIRT in PD.

Furthermore, our results showed robust alterations in evoked oscillatory power following TMS over M1 by MIRT. Numerous studies have confirmed that abnormal cortical oscillations are a hallmark of PD. Beta band (14-30 Hz) activity in cortico-basal ganglia circuits is broadly associated with static motor control, such as tonic or postural contractions. Theta oscillations (4-7 Hz) are associated with resting tremors, and their relative power was increased significantly in PD subjects (Bosch et al., 2021). Gamma rhythm (>30 Hz) abnormalities in the motor cortex are important in the generation of motor symptoms in PD (Hanajima et al., 2014). Theta and gamma connectivity within fronto-temporo-parietal networks have been shown to be associated with affective and cognitive symptoms in PD (Iyer et al., 2020). After MIRT, our results showed no significant differences in oscillatory power across the whole brain but revealed theta (4-7 Hz), beta (13-30 Hz) and gamma (30-45 Hz) band power reductions in the central and parietal regions. Only the gamma band power of the parieto-occipital region decreased in non-responders. These findings support the results of previous interventions for PD showing that transcranial alternating current stimulation (tACS) (Del Felic et al., 2019) and the use of gait compensation strategies (Tosserams et al., 2022) to improve PD motor symptoms were closely related to decreased beta oscillation. Only one previous small-sample study mentioned a significant change in beta modulation levels after MIRT (Marchesi et al., 2019). As marked changes in oscillatory power were observed following PD therapy, our study found that beta power was significantly associated with motor symptom (UPDRS III) enhancement and that gamma power was related to cognitive performance (MMSE) after MIRT in PD. Consistent with the clinical characteristics, main motor functions were enhanced in responders, and mainly the non-motor symptoms were improved in non-responders, which corresponds to the reduced gamma power after MIRT. However, the oscillatory power significantly affected by MIRT was not significantly correlated with the quality-of-life assessment after 3 months of follow-up (PDQ-39). The main reason is that the assessment of neural activity is collected only after MIRT and lacks follow-up results. Therefore, our findings suggest that beta oscillatory power modulation contributes to the benefits of motor function, and the gamma band might be involved in non-motor improvements by MIRT in PD.

Several limitations should be considered. A control group for MITR was not established. However, inclusion in the placebo group is difficult because PD patients receiving MIRT require prolonged hospitalization on an individual basis due to complex symptoms. Therefore, the traditional rehabilitation method can be selected as the positive control group. Given that multiple brain regions are abnormally involved in the basal ganglia-thalamus-cortex circuit, although the primary motor cortex (M1) plays an important role in PD, stimulation of other target brain regions (such as the DLPFC and SMA) with single-pulse TMS can further verify whether MIRT treatment has a specific effect on M1 macrophages and reveal more subtle aspects of the PD pathological mechanism and clinical response to treatment.

## Conclusion

This is the first study to use concurrent TMS and EEG to explore the benefit of MIRT in PD. We found key changes in cortical and oscillatory activity following TMS over M1 by MIRT, with a lower GMFP and reductions in beta and gamma power being associated with motor and non-motor clinical responses. Our findings provide important support for the effectiveness of MIRT clinical research and provide potential targets for clinical PD treatment.

## Data availability statement

The raw data supporting the conclusions of this article are available through the corresponding authors.

## Ethics statement

The studies involving human participants were reviewed and approved by the Ethics Committee of Beijing Rehabilitation Hospital, Capital Medical University (2020bkky010). The patients/participants provided their written informed consent to participate in this study. Written informed consent was obtained from the individual(s) for the publication of any potentially identifiable images or data included in this article.

## Author contributions

GP wrote the manuscript. GP, XL, and QH analyzed the data. GP, XL, and ZS interpreted the data. LW, DS, SF, JW, and JZ revised the manuscript. BF designed and supervised the experiments. All authors contributed to the article and approved the submitted version.
