# Supplementary material for: Characterizing cortical responses to short-term multidisciplinary intensive rehabilitation treatment in patients with Parkinson’s disease: A transcranial magnetic stimulation and electroencephalography study
Source: Front Aging Neurosci. 2022 Nov 3;14:1045073. doi: 10.3389/fnagi.2022.1045073 (PMC9669794; doi:10.3389/fnagi.2022.1045073)
Supplement: Supplementary file 1 [file Table_1.docx]

Supplementary Material

# Supplementary Table

**Supplementary TABLE 1** Baseline clinical characteristics of the study.

| PD Subjects | | Responding Group (N=22) | Nonresponding Group (N=26) |  |
| --- | --- | --- | --- | --- |
|  |  | Baseline  Mean (SD) | Baseline Mean (SD) | p-value |
| Subject Demographic | Sex (male/female) | 13/9 | 11/25 | 0.252^a^ |
|  | Age (years) | 62.64(7.22) | 61.35(6.97) | 0.533^b^ |
|  | H-Y Stage | 2.34(0.52) | 2.33(0.42) | 0.955^a^ |
|  | Disease Duration(months) | 88.14(55.07) | 78.18(41.75) | 0.423^a^ |
|  | LEED (mg) | 636.93(356.83) | 495.40(242.80) | 0.111^b^ |
|  | BMI | 23.40(2.84) | 23.86(3.32) | 0.611^b^ |
|  | Education (years) | 11.93(4.52) | 11.81(4.10) | 0.921^b^ |
| Motor Symptoms  Assessment | MDS-UPDRS III | 43.09(10.88) | 37.12(10.62) | 0.061^b^ |
|  | Tremor | 4.46(4.00) | 3.77(3.67) | 0.643^a^ |
|  | Bradykinesia | 21.82(7.10) | 19.08(6.12) | 0.158^b^ |
|  | Rigidity | 9.86(1.98) | 8.89(1.73) | 0.074^b^ |
|  | Axial | 6.96(3.74) | 5.39(2.58) | 0.103^a^ |
|  | M-PAS | 49.68(6.85) | 51.54(5.27) | 0.260^a^ |
|  | FTSTS | 10.89(2.37) | 10.73(2.56) | 0.825^b^ |
|  | TUG | 9.75(2.24) | 10.90(5.39) | 0.869^a^ |
|  | BBS | 22.68(3.56) | 23.42(2.27) | 0.746^a^ |
|  | 10MW-Com | 1.25(0.22) | 1.14(0.25) | 0.162^a^ |
|  | 10MW-Fast | 1.74(0.29) | 1.56(0.35) | 0.104^a^ |
|  | 6MWD | 448.32(112.85) | 436.46(87.54) | 0.246^a^ |
| Non-motor Symptoms Assessment | MMSE | 27.09(2.67) | 27.31(2.88) | 0.592^a^ |
|  | MoCA | 24.95(3.33) | 25.08(4.26) | 0.655^a^ |
|  | Stroop Test | 45.09(17.07) | 50.27(4.60) | 0.835^a^ |
|  | PASAT | 23.50(16.58) | 21.50(14.95) | 0.662^b^ |
|  | PDQ-39 | 24.14(10.12) | 24.03(9.16) | 0.844^a^ |

Values are mean (SD).

SD= Standard Deviation; LEED=levodopa equivalent dose; BMI= Body Mass Index; MIRT=Multidisciplinary intensive rehabilitation treatment; MDS-UPDRS III=Movement Disorders Society-Sponsored Revised Unified Parkinson's Disease Rating Scale part III; M-PAS=Modified Parkinson's Activity Scale; BBS=Berg Balance Scale; FTSTS=Five Times Sit to Stand; TUG=Timed Up and Go; 10MW=10-Meter Walking; 6MWD=6-Minute Walking Distance; MMSE=Mini Mental State Examination; MoCA=Montreal Cognitive Assessment; PASAT=Paced Auditory Serial Addition Task; PDQ-39=39-Item Parkinson’s Disease Questionnaire.

**^a^** Mann-Whitney *U* tests.

**^b^** Independent samples *T*-tests.
